# Visualization analysis for emotional characteristics of autism spectrum disorder from cinemetrics perspective

**DOI:** 10.3389/fpubh.2025.1608608

**Published:** 2025-10-31

**Authors:** Mengyuan Shen, Yawen Jing, Qingyuan Liu, Chen Li, Ning Xu

**Affiliations:** ^1^School of Art & Design, Liaoning Petrochemical University, Fushun, China; ^2^College of Medicine and Biological Information Engineering, Northeastern University, Shenyang, China

**Keywords:** visualization analysis, emotional characteristics, autism spectrum disorder, cinemetrics, statistical significance

## Abstract

The attention paid to *Autism Spectrum Disorder* (ASD) in film art and audiovisual communication has promoted the popularization of ASD knowledge and the development of treatment and education measures. As society pays more attention to ASD research and education, the limitations of traditional qualitative research methods are gradually becoming apparent, particularly in the dynamic and nuanced quantification of emotional characteristics, which hinders the practical application of research results. As an emerging research paradigm, cinemetrics provides new perspectives for film research. In this paper, 20 Chinese autism-themed films with 2,627 shots are selected and statistically analyzed in terms of style, rhythm, and space for their emotional character clips. *Average Shot Length* (ASL) and *Median Shot Length* (MSL) are compared using a Paired Samples *t*-test (*t* = 5.620, *p* < 0.001) to verify the statistical significance of rhythmic differences. The results indicate that the emotional characteristics of autistic individuals in various films differ significantly in terms of shot length, editing rate, camera movement, and composition. It is found that ASL values are consistently higher than MSL values indicates a systematic rhythmic pattern rather than random fluctuation, providing a reliable quantitative basis for further analysis. These quantitative analyses provide objective data support for the study of the emotional characteristics of ASD, and also offer potential references for practical applications such as expression recognition training and movement regulation programs. This paper can help the public to understand the emotional state of ASD people and open up new paths for future investigation of ASD intervention.

## 1 Introduction

Individuals with *Autism Spectrum Disorder* (ASD) are characterized by social challenges and stereotyped repetitive behaviors, which significantly impact their learning and quality of life. In recent years, as societal awareness of ASD has increased, relevant policies in China have gradually improved. Policies related to ASD research and education have advanced, and targeted interventions have been progressively developed. In 2022, the General Office of the State Council forwarded the “14th Five-Year Plan for the Development and Enhancement of Special Education,” (1) jointly formulated by the Ministry of Education and other departments. This plan clearly states that special education will be developed during the 14th Five-Year Plan period and outlines the need for a rationally planned layout of special education schools for ASD children. It advocates for the establishment of specialized special education schools for ASD in provincial capitals, cities with single-listed plans, and larger cities, aiming to improve the currently weak foundation of ASD individuals with ASD. In the same year, China launched two batches of brain science programs (2), focusing on strengthening the diagnostic and intervention capacity for ASD individuals and other childhood brain disorders. These programs set specific goals to improve diagnostic efficiency. These measures mark a significant advancement in ASD research and policy support in China, demonstrating the commitment from the government to provide scientific diagnosis and effective support for ASD individuals.

In 2024, the Rehabilitation Department of the China Disabled Persons’ Federation (CDPF) launched the “Implementation Plan for the Care and Promotion of Actions for Children with Autism Spectrum Disorders (2024–2028),” (3) further emphasizing the high level of commitment from the government to ASD research and support. This policy not only intensifies the focus on ASD individuals but also emphasizes the provision of education and intervention services. It reflects the shift of government from an initial focus on special education to the creation of a more systematic and scientific intervention system. The implementation of these policies has enhanced early diagnosis, intervention, and education for ASD individuals, promoting the development of related research. Especially, due to communication disorder is a common feature for ASD individuals, it is crucial for doctors and rehabilitation therapists to accurately interpret ASD individuals’ emotional characteristics during diagnosis, intervention, and education. Therefore, the analysis of patients’ expressions and body language becomes a key aspect in relevant research and clinical practice.

In the research domain of ASD individuals, the application of expression analysis in emotion recognition receives more and more attention (4, 5). In particular, micro-expressions consist of 39 fragmented action units, and they only display certain components of fully expressed expressions (6, 7). Therefore, analyzing expressions is highly effective for emotional analysis of ASD individuals, and various interventions and training methods have been proposed to improve emotion recognition in ASD individuals. For example, the work of (8, 9) indicates that ASD individuals, both children and adults, often struggle to recognize the expressions of others, significantly impairing their ability to engage in social interactions effectively.

Besides, expression analysis in emotional analysis of ASD individuals, body language analysis is also an important respect for identifying the emotional characteristics of ASD individuals. This involves interpreting emotional signals conveyed through body posture, spatial positioning, body configuration, and movement patterns (10, 11). Intense emotions often trigger full-body movements, such as curling up when sad, jumping when joyful, clapping when excited, swaying when anxious, or spinning when engaging in self-stimulatory behavior (12, 13). Therefore, understanding the body language of ASD individuals is one of the critical technical approaches to discern their emotional characteristics. For example, automated video analysis technologies can be applied to objectively quantify these social movement markers, aiming to enhance the accuracy and reproducibility of assessments (14).

As society’s understanding of ASD individuals deepens, the portrayal of emotional characteristics of ASD individuals in films has attracted widespread attention, where expression and body language analysis play an important role. Traditional film studies primarily rely on qualitative analysis, which often fails to capture the subtle emotional changes of individuals with ASD. The introduction of cinemetrics, however, provides a new perspective and method for quantitatively analyzing the emotional features in autism-themed films (15). In this process, *Average Shot Length* (ASL) and *Median Shot Length* (MSL) are commonly used quantitative metrics in image analysis. ASL represents the average duration of the shots in a film, while MSL is determined by sorting the shot durations and selecting the middle value to represent the central tendency of the shot lengths. By conducting statistical analysis of these shot lengths, we can capture the emotional rhythm and fluctuations of the ASD characters in related films. Therefore, cinemetrics method can break through the subjectivity and limitations of traditional film analysis, allowing for comparative emotional analysis across films and revealing systematic patterns in emotional expression across different autism-themed films.

Additionally, traditional methods lack a dynamic and intuitive visualization of emotional characteristics, limiting the practical application of research findings. In response to these challenges, cinemetrics employs tools such as artificial intelligence, big data, and data mining to contribute to related research domains. Hence, cinemetrics can introduce a shift in the conceptual framework and research paradigm in film studies (15), offering a fresh perspective on the emotional characteristics of ASD individuals. In this paper, 20 representative samples with 2,627 shots are selected from 37 Chinese autism-themed films to conduct cinemetrics analysis, aiming to reveal the unique emotional characteristics to provide a new quantitative and objective research and intervention for ASD individuals. Based on this experimental design, our research question is: Do Chinese autism-themed films exhibit a systematic rhythm pattern (ASL > MSL), thereby reflecting a unique portrayal of the emotional characteristics of individuals with ASD? To find the answer to this question, we hypothesize that Chinese autism-themed films can exhibit systematic differences between ASL and MSL, with ASL values consistently exceeding MSL; this disparity is expected to reflect distinctive emotional characteristics and rhythmic features associated with ASD individuals portrayals. If ASL was generally higher than MSL, it may suggest that these films exhibit an inherent consistency in emotional expression rather than random fluctuations. Verifying this hypothesis will not only provide a new quantitative analysis method in this paper of emotional characteristics in autism-themed films, but also offer theoretical support for interventions and education for individuals with ASD. Therefore, this paper integrates emotional analysis and cinemetrics to propose a quantitative analysis of emotional characteristics in autism-themed films.

## 2 Related work

### 2.1 ASD emotional characteristics

ASD is a neurodevelopmental condition characterized by deficits in social interaction and communication, along with repetitive and stereotyped patterns of behavior. Individuals with ASD often experience a range of emotional and behavioral challenges, including mood disorders such as irritability, anxiety, depression, and instances of verbal aggression, physical aggression, or disruptive behaviors (16). According to the “Chinese Autism Spectrum Disorder Screening for Children Aged 0 to 6 Years,” the prevalence of ASD among Chinese children aged 0 to 6 is approximately 1.8% (17). ASD Children typically struggle with understanding their psychological states as well as those of others, making it difficult for them to integrate and interpret social and emotional information effectively. This lack of theory of mind ability directly impacts their communication and socialization skills (18). Recognizing emotional cues is a fundamental psychological and social competence that forms the basis for successful social interactions. For most children, emotion recognition is a critical social skill that develops during early childhood (19). From a developmental psychology perspective, an ability of individual to recognize emotions improves with age, experience, and cognitive development.

Emotion regulation can be categorized into five main modalities, of which cognitive reappraisal and expressive suppression are the two most common (20). This paper categorizes these strategies as adaptive emotion regulation (e.g., cognitive reappraisal and acceptance) and maladaptive emotion regulation (e.g., avoidance and inhibition) base on their association with mental health status (21). Compared to their typically developing peers, children and adults with ASD appear to utilize adaptive emotion regulation strategies less frequently (22), while employing maladaptive emotion regulation strategies significantly more frequently (23). In addition, individuals with ASD tend to have more fragmented and incoherent emotion regulation strategies (24). Overall, individuals with ASD are dysfunctional in emotion regulation and exhibit difficulties in emotion regulation at different stages of growth, which is closely related to their lack of ability to select and use adaptive emotion regulation strategies.

Specifically, individuals with ASD typically show less use of adaptive emotion regulation strategies and more frequent reliance on maladaptive emotion regulation strategies. Children and adolescents with ASD individuals tend to favor simple coping styles, such as venting or avoidance, and less use of more complex regulation strategies when confronted with emotional problems; adults with ASD individuals are more likely to rely on maladaptive emotion regulation strategies, such as inhibition. In addition, flaws in theories of mind may lead to difficulties for individuals with ASD in assessing their psychological states, further weakening their ability to regulate their emotions.

### 2.2 Development of cinemetrics

In the traditional research of films, the analysis of emotional characteristics always relies on the personal viewing experiences of researchers. Such approaches are heavily dependent on individual subjective judgment, lacking unified quantitative standards and reproducible analytical frameworks, and are unable to provide data that can be objectively verified (25). These limitations constrain both the scientific rigor of research conclusions and the validity of cross-film comparisons.

To address the limitations of the traditional research methods, Barry Salt first introduced the concept of “cinemetrics” to objectively and quantitatively analyze films, which can incorporate big data analytics and visualization techniques into its methodological framework. The discipline measures and analyzes film styles through systematic and digital means, and the process covers the calculation of formal elements or variables that can reflect film styles. Cinemetrics provides a more objective, systematic, and precise mode of film analysis, which can be seen as its significant advantage (26). It deepens the exploration of the artistic and narrative characteristics of the film. Salt introduced a scientistic viewpoint in film studies, advocating the quantitative study of formal elements of film as objective data. He advocated that film studies should start from a film itself rather than relying on external theories. He drew many conclusions about the artistic and narrative characteristics of film by measuring data such as the length of shots of a film and the movement of the camera.

In recent years, Chinese scholars have shown a growing interest in cinemetrics. Daoxin Li points out that cinemetrics addresses the issue of “detachment from the essence of cinema and a bias toward perceptual experience” in current film research. Furthermore, he suggests that it can significantly promote a shift in research thinking and the renewal of paradigms in film studies, particularly in the study of contemporary Chinese cinema (27). The emergence and development of cinemetrics provide a novel perspective and methodology for film research, opening new thoughts for the application of digital humanities in cinema studies. As disciplines become increasingly integrated and with the rapid advancement of big data and artificial intelligence, the scope of cinemetrics continues to broaden, establishing it as one of the leading theories in the field of digital humanities. In this context, Salt not only deepens our understanding of the core principles of cinemetrics but also offers valuable insights for developing film theory.

By conducting precise measurements and quantitative modeling across multiple dimensions such as shot length, editing rhythm, color saturation, musical frequency characteristics, and changes in expression, cinemetrics substantially enhances the objectivity and systematic nature of film research. It enables the generation of visualized emotional fluctuation curves and reveals precise correspondences between emotional expression and narrative structure. Especially in the study of autism-themed films, cinemetrics can not only identify subtle emotional variations that traditional analytical methods struggle to capture, but can also construct a set of comparable emotional parameters across multiple films. Therefore, cinemetrics can provide a robust data foundation for exploring similarities and differences in how various directors portray the emotional characteristics of individuals with ASD (28).

In addition to the technical advantages of cinemetrics, it can also emphasize data sharing and interdisciplinary integration in related research fields (29). Through open cinemetrics analysis platforms, communication barriers between academia and the public have been mitigated, gradually dismantling the insular academic ecosystem that once emerged from overemphasis on intellectual property protection. This collaborative research mechanism not only expands academic perspectives but also accelerates the dissemination and application of research findings across a wider spectrum of public health. Furthermore, it can foster the development of a film research community anchored in data-driven collaboration (30).

### 2.3 Visual analysis of ASD characters in films

The portrayal of ASD individuals in films and other media has become an important area of academic research. Studies indicate that films, as cultural media, can enhance public awareness and understanding of ASD individuals, but they can also perpetuate negative stereotypes and misconceptions. In their systematic review of ASD individuals representations in fictional media, Jones et al. (25) argue that while depictions of ASD individuals in films contribute to raising public awareness of the disorder, these portrayals often rely excessively on characters’ exceptional talents, while overlooking the everyday challenges faced by individuals with ASD. For instance, while the film *Rain Man* brought increased public attention to ASD individuals, the characters depicted often present individuals with ASD as possessing extraordinary abilities, leading to societal misconceptions and the stereotyping of the capabilities of people on the autism spectrum.

Hungerford also points out that while ASD individuals portrayals in film can help raise public awareness (31), their negative impact lies in the overemphasis on the “alienating” characteristics of ASD individuals, which often fail to accurately depict the real-life experiences of individuals with ASD in terms of social, emotional, and behavioral aspects. This limitation in representation calls for future media works to offer more diverse and empathetic portrayals of ASD individuals. Additionally, Dean and Nordahl-Hansen conducted a review of ASD individuals representations in film and television (32), arguing that a significant gap remains in the depiction of ASD individuals characters in current research. They suggest that a more interdisciplinary and diverse approach is needed to enhance the social acceptance and understanding of ASD individuals.

With the rise of *image metrics* as a quantitative film analysis method, film studies have begun shifting from traditional qualitative analysis to data-driven, quantitative research. Image metrics employs statistical tools to analyze elements such as shot length, editing rhythm, and camera movement, providing a more systematic and objective framework for analysis. Ji et al. (33) proposed a new facial action unit dataset and analyzed the expression characteristics of children with ASD individuals. The results indicated that children with ASD individuals exhibit more irregular and diverse expression patterns, offering new data support for image-based emotional analysis. This paper highlights how film, as a medium for emotional expression, conveys the emotional characteristics of individuals with ASD through visual language, including shots, editing, and expressions.

In recent literature, several works have explored the cognitive and emotional aspects of ASD individuals, such as the executive functions and emotional regulation in individuals with ASD. Guazzo and Ginolfi’s research (34) highlights the crucial role of executive functions in the development of emotional regulation and how deficits in these functions often lead to difficulties in social cognition and behavior in ASD individuals. This understanding is integral in the portrayal of ASD individuals in the media, as it can help shape more realistic and nuanced representations. Furthermore, studies by Fernandes et al. (35) in the realm of social cognition and theory of mind have pointed to key differences in cognitive processing between ASD and other disorders, which can influence how characters with ASD individuals are depicted in films and how viewers interpret their actions. Additionally, Peterson’s study (36) on theory of mind and empathic behavior in children with ASD individuals further informs the emotional and social challenges faced by individuals on the autism spectrum, contributing to more empathetic portrayals in media.

## 3 Research data and methods

### 3.1 Research data

Due to significant differences between the Eastern and Western world in history, culture, economy, politics, religion, and other aspects, research in the field of film studies is often conducted based on some basic areas, including China, Hollywood (the United States), Europe and Australia, Bollywood (India), Japan and South Korea, and other countries and regions. Particularly, we take Chinese films as an initialization to carry out a series of data collection and preparation work. Currently, this paper utilizes data based on Chinese autism-themed films, and we are in the process of preparing data on Hollywood and European films.

In this paper, 20 Chinese autism-themed films released between 2008 and 2024 are selected for analysis. To ensure diversity in the research subjects, five microfilms and 15 feature-length films are included^1^. Microfilms typically convey condensed emotions and ideas in a concise format, while feature-length films allow for more complex plot development and character exploration. By analyzing both types of films, this paper provides a comprehensive understanding of how ASD individuals are represented across different film formats and reveals the unique perspectives and expressive techniques of various genres in depicting ASD individuals. Furthermore, to ensure the accuracy of the experimental results, ASD-related emotional clips from each film are manually selected and analyzed. Based on these 20 selected films and video segmentation for emotional clips, finally, we obtain 106 emotional clips for our cinemetrics analysis, including a total of 2,627 shots.

In this paper, the selection of films followed strict inclusion and exclusion criteria, including three inclusion criteria and two exclusion criteria as follows.

Inclusion-Rule-I: the films must be produced in the People’s Republic of China, including the Chinese Mainland, Hong Kong, Macau, and Taiwan.

Inclusion-Rule-II: the films must have been released between 2008 and 2024.

Inclusion-Rule-III: film types include feature films and microfilms, but documentaries are not included.

Exclusion-Rule-I: there are some films with ASD characters, but if the focus of the film was not on ASD characters or if the portrayal of ASD was insufficient, then those films are excluded.

Exclusion-Rule-II: some films or microfilms are excluded due to weaker production techniques, lack of artistic expression, simplistic plotlines, or insufficient depth in analysis.

**Figure 1:**
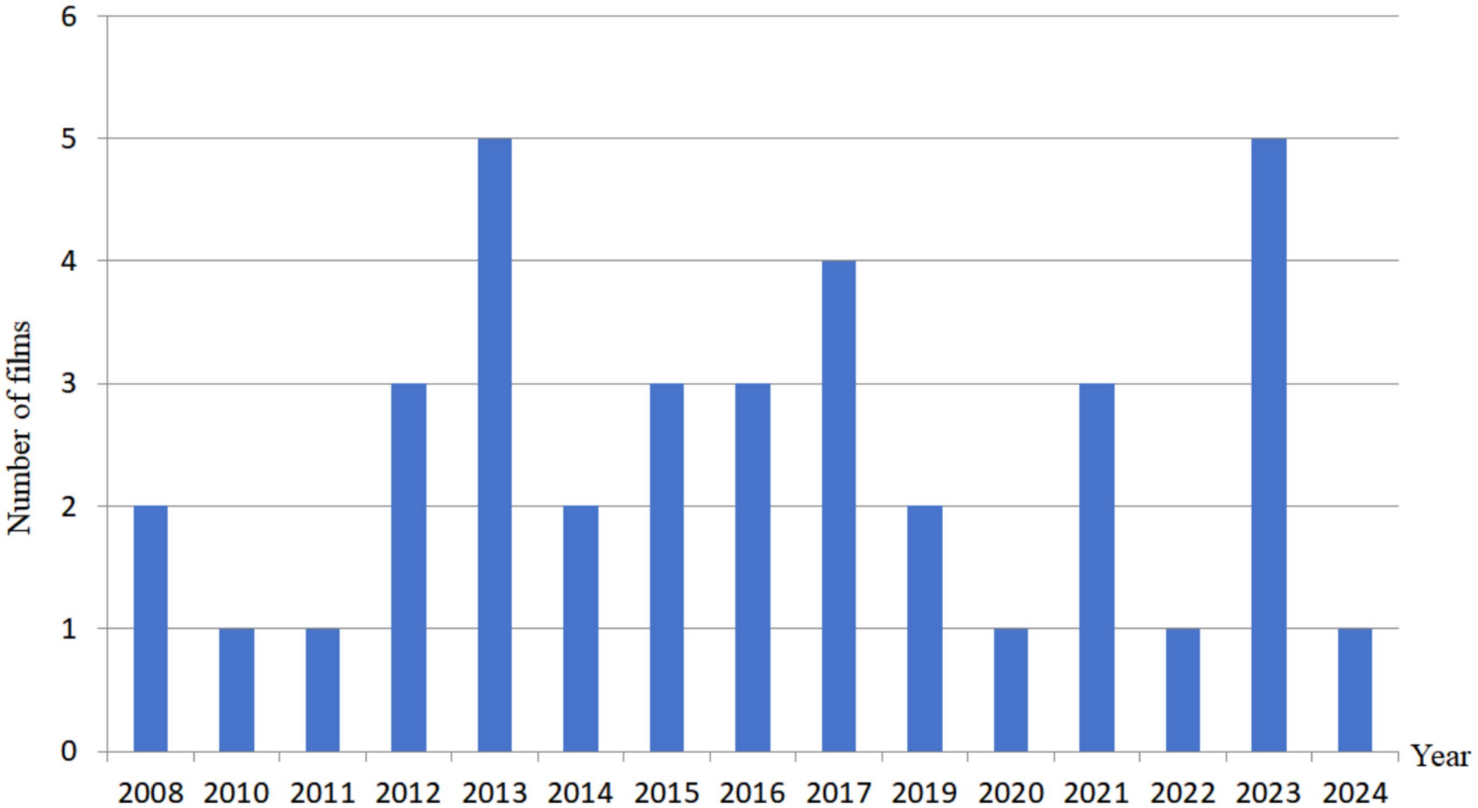
Number of films related to ASD from 2008 to 2024.

Based on these five rules above, a total of 37 relevant films are finally identified after a careful screening, and after further strict selection, 20 films are ultimately selected in this paper. The number of films related to ASD from 2008 to 2024 is shown in Figure 1, where the longitudinal dimension is explored.

From Figure 1, we can find that the number of Chinese autism-themed films is relatively limited. As of 2024, only 37 films meet the predefined inclusion criteria, where the details are shown in Table 1. Due to the total number of these films is small, we do not set a fixed ratio or a constant to limit the selection of film data in this paper. Instead, we apply a traversal strategy to go through all these 37 films, where we can retain the maximum amount of valid data. Based on this traversal strategy, 20 films are finally selected for analysis in this paper, including 15 feature films and 5 microfilms. Table 1 shows film data selection process in this paper.

**Table 1:** Film data selection from 37 Chinese autism-themed films from 2008 to 2024.

Film ID	Film title	Director	Production area	Release time	Film length type	Inclusion rules			Exclusion rules		Finally included in the analysis of this paper
						I	II	III	I	II	
1	*Ticket*	Zhiliang Zhang	Mainland, Hong Kong	2008	Feature Film	✓	✓	✓	✗	✗	Yes
2	*Do not Say Sorry Do not Say Bye*	Jiankui Liu	Mainland	2008	Feature Film	✓	✓	✓	✗	✗	Yes
3	*Ocean Heaven*	Xiaolu Xue	Mainland, Hong Kong	2010	Feature Film	✓	✓	✓	✗	✗	Yes
4	*Because, So*	A Dai	Mainland	2011	Microfilm	✓	✓	✓	✓	✓	No
5	*Nymph*	Yiheng Nie	Mainland	2012	Microfilm	✓	✓	✓	✓	✓	No
6	*Son of the Stars*	Miao Chen	Mainland	2012	Feature Film	✓	✓	✓	✗	✗	Yes
7	*Singing Fish*	Shu San	Mainland	2012	Feature Film	✓	✓	✓	✗	✗	Yes
8	*Smell Color*	Xiubo Hao	Mainland	2013	Microfilm	✓	✓	✓	✗	✗	Yes
9	*Tears from the Stars*	Hongye Liu	Mainland	2013	Microfilm	✓	✓	✓	✓	✓	No
10	*My Running Shadow*	Gangliang Fang	Mainland	2013	Feature Film	✓	✓	✓	✓	✓	No
11	*Colorless Dream Flower Flying*	Zheng Ma	Mainland	2013	Feature Film	✓	✓	✓	✓	✓	No
12	*On the Other Shore Bloom of Flowers*	Jia Qi	Mainland	2013	Microfilm	✓	✓	✓	✗	✗	Yes
13	*Love in My Home*	Feng Wang	Mainland	2014	Microfilm	✓	✓	✓	✓	✓	No
14	*The Guardian of the Stars*	Bo Zhu	Mainland	2014	Microfilm	✓	✓	✓	✓	✓	No
15	*Fate of Love*	Tao Shi	Mainland	2015	Microfilm	✓	✓	✓	✗	✗	Yes
16	*Open Your Heart*	Zhen Yang	Mainland	2015	Feature Film	✓	✓	✓	✗	✗	Yes
17	*Angels Are Not Alone*	Wei Wei	Mainland	2015	Feature Film	✓	✓	✓	✓	✓	No
18	*Heart Disease*	Bin Li	Mainland	2016	Feature Film	✓	✓	✓	✓	✓	No
19	*Destiny*	Wei Zhang	Mainland	2016	Feature Film	✓	✓	✓	✗	✗	Yes
20	*A Horse with Hope*	Haibin Ba	Mainland	2016	Feature Film	✓	✓	✓	✓	✓	No
21	*Tomorrow Is Another Day*	Dali Chen	Mainland, Hong Kong	2017	Feature Film	✓	✓	✓	✗	✗	Yes
22	*Whose Baby Am I*	Nan Li	Mainland	2017	Feature Film	✓	✓	✓	✗	✗	Yes
23	*The Seventeenth Abandonment*	Zhuolin Li	Mainland	2017	Microfilm	✓	✓	✓	✗	✗	Yes
24	*Ming and Ming*	Haobo Hu	Mainland	2017	Feature Film	✓	✓	✓	✗	✗	Yes
25	*Yesterday Once More*	Qidong Zhang	Mainland	2019	Feature Film	✓	✓	✓	✗	✗	Yes
26	*Your Beauty*	Li Xu	Mainland	2019	Feature Film	✓	✓	✓	✗	✗	Yes
27	*The Sea and Llight Rain*	Yi Dong	Mainland	2020	Microfilm	✓	✓	✓	✓	✓	No
28	*Over*	Xiuchun Kou	Mainland	2021	Feature Film	✓	✓	✓	✓	✓	No
29	*Walking to the Moon*	Yangqing Chen	Mainland	2021	Microfilm	✓	✓	✓	✗	✗	Yes
30	*The Music of Breathing*	Anqing Wang	Mainland	2021	Feature Film	✓	✓	✓	✓	✓	No
31	*Like A Stone*	Chenglin Chou	Mainland	2022	Microfilm	✓	✓	✓	✓	✓	No
32	*He Is My Brother*	Xiuzhuan Guo	Mainland	2023	Feature Film	✓	✓	✓	✗	✗	Yes
33	*Love Without Words 2*	Yizhi Tan	Mainland	2023	Feature Film	✓	✓	✓	✓	✓	No
34	*Do not Call Me God of Gamblers*	Yaoming Pan	Mainland, Hong Kong	2023	Feature Film	✓	✓	✓	✓	✓	No
35	*Little Girl Like A Sunflower*	Yingqi Chen	Mainland	2023	Feature Film	✓	✓	✓	✗	✗	Yes
36	*Dream Forest*	Chao Fan	Mainland	2023	Feature Film	✓	✓	✓	✗	✗	Yes
37	*The Hedgehog*	Changwei Gu	Mainland, Hong Kong	2024	Feature Film	✓	✓	✓	✓	✓	No

If a film met the inclusion and exclusion rules, then a “✓” is given in this table; otherwise, a “✗” is given. So, if a film obtained three “✓” in inclusion rules and two “✗” in exclusion rules, then it can be finally included in the analysis of this paper.

### 3.2 Research methods

In this paper, shot metrics and video segmentation are used as the primary research methods, with ASL and MSL as the key statistical indices in cinemetrics analysis. ASL is calculated by dividing the total length of the film by the total number of shots, providing an average duration for the shots. MSL is determined by dividing all shots in the film by their duration, then selecting the length of the shot in the middle of the distribution range after sorting them by duration. This metric represents the central tendency of shot durations.

In film analysis, shot length measurements often depend on video segmentation techniques that accurately identify and measure the duration of each shot. These statistics allow film researchers to analyze the editing pace and narrative style of the film, as well as the creativity of the director. In this paper, Adobe Premiere Pro 2021 is applied as the video editing tool, specifically its scene detection feature, to statistically analyze the shot segmentation of the 20 selected autism-themed films. The data in this paper were jointly prepared by the first author and the corresponding author from the research field of media science, along with an author from the field of biomedical engineering. Our data labeling process includes three quality control stages:

Stage-I: The first author independently performs an initial annotation, forming the first version of data.

Stage-II: The corresponding author and first author jointly check and revise all data, resulting in the second version of data.

Stage-III: The first author, corresponding author, and biomedical engineering author collaboratively conduct a final verification and calibration for the third version of data.

**Figure 2:**
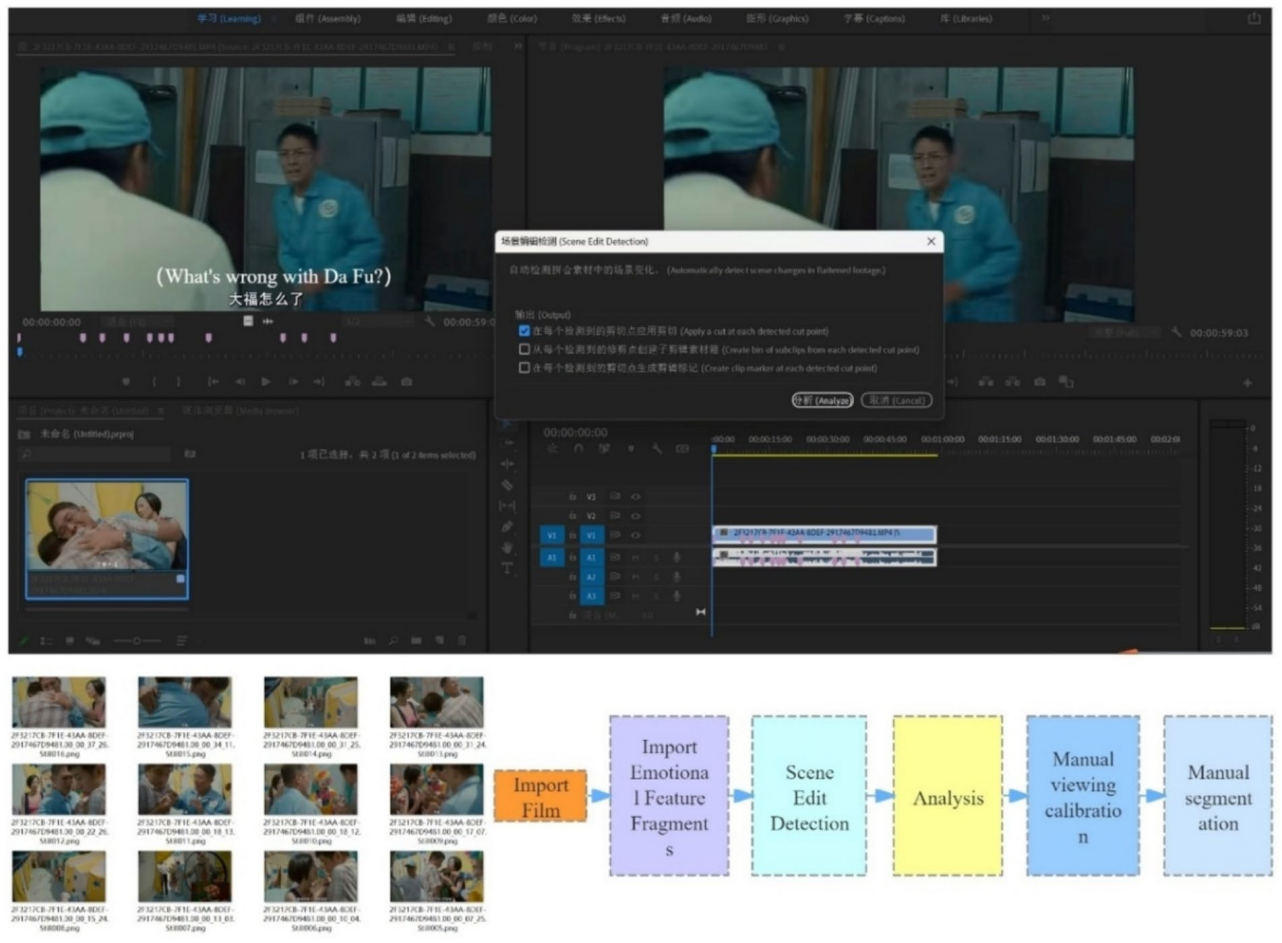
Data preparation workflow in this paper.

Specifically base on these three quality control stages above, we apply both automatic segmentation and subsequent manual calibration functions in Adobe Premiere Pro 2021 to ensure our data preparation quality. The data preparation workflow is shown in Figure 2.

As the data preparation workflow in Figure 2, the finalized emotional feature segments are input into the Adobe Premiere Pro 2021 software. After selecting the relevant segments, automatic analysis is performed using the “Scene Edit Detection” function. Upon completion of the analysis, we conduct a manual review and observation of the segments, repeatedly calibrating and manually segmenting as necessary to ensure the accuracy and reliability of the data annotation.

In analyzing transitions such as dissolve, fade-ins, and fade-outs common in early films, we find that these special effects posed challenges for the automatic shot segmentation function of Adobe Premiere Pro 2021. To address this, this paper first employs the automatic segmentation technique to perform an initial shot division. Based on this preliminary segmentation, detailed manual verification and correction steps are then carried out to ensure data accuracy and the reliability of the results. By combining automatic and manual segmentation, our work in this paper can thoroughly explore the shot length characteristics of emotional clips featuring ASD individuals patients, providing new insights and data to enhance understanding of how individuals with ASD are portrayed in films.

## 4 Visualization and analysis for emotional characteristics of autism-themed films

Christian Metz from France was the first significant figure to introduce structuralist theory into the field of film studies. In 1964, he applied concepts from structuralist linguistics to film analysis, thereby laying the foundation for structuralist film theory (37). This approach systematically examines the symbolic and textual structure of films and explores their narrative style. However, traditional research on films often relies on qualitative and descriptive methods, which are subjective and lack a systematic and objective framework. These methods struggle to capture and quantify the detailed differences of emotional expression in films and fail to provide a dynamic and intuitive visual representation of these emotional characteristics. As a result, their applicability and practical value are limited. In this context, the integration of data visualization in cinemetrics offers a more objective and quantitative approach, making the analysis of emotional characteristics both dynamic and intuitive, and thus enhancing the practical value of film research.

### 4.1 Lens length distribution based on film style analysis

In the lens length distribution-based analysis of film styles, ASL and MSL are two important indices usually used. ASL is a widely used statistical index in film studies to calculate the average duration of all shots in a film. It is obtained by dividing the total duration of the film by the number of shots, while MSL is obtained by sorting all shots by duration and then choosing the middle value to represent the central tendency of the distribution of shots. Since ASL is susceptible to extreme values, while MSL can more accurately reflect the actual distribution characteristics of shot lengths. The use of both ASL and MSL in analyzing film styles makes the data analysis comprehensive and detailed.

**Figure 3:**
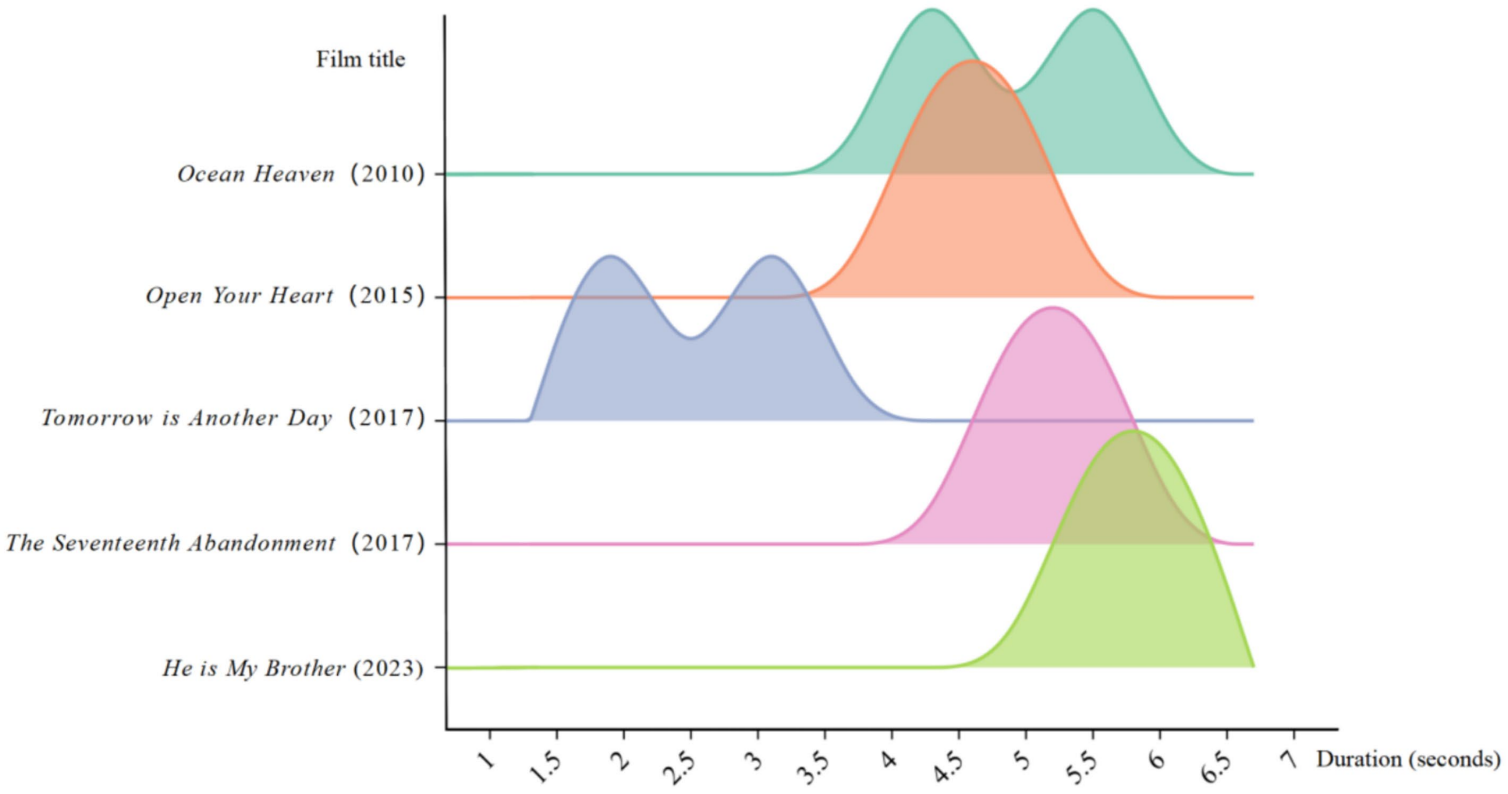
Comparison of ASL and MSL in the emotional characteristics of clips of autism-themed films.

Based on ratings from platforms such as the Chinese Cinema Knowledge System, China Film Network, IMDB, and other online film databases, and concerning the popularity of the films, five representative films are selected as samples in this paper: *Ocean Heaven*, *The Seventeenth Abandonment*, *Tomorrow is Another Day*, *Open Your Heart*, and *He is My Brother*. This paper statistically derived shot length and shot count data, which are used to create the “Comparison of various shot length values in emotional characteristics clips of autism-themed films” (Table 2) and the “Comparison of ASL and MSL in emotional characteristics clips of autism-themed films” (Figure 3).

**Table 2:** Comparison of various shot length values in emotional characteristics clips of autism-themed films, where “min” and “sec” represent “minute” and “second,” respectively.

Film title	*Ocean* *Heaven* (2010)	*Open Your Heart* (2015)	*The Seventeenth Abandonment* (2017)	*Tomorrow Is Another Day* (2017)	*He Is My Brother* (2023)
Clip duration (min: sec)	24:28	2:01	11:32	15:02	20:32
Number of lenses	261	184	22	220	210
ASL (sec)	5.6	4.9	5.5	3.1	5.9
MSL (sec)	4.0	4.0	5.0	2.0	4.0
Minimum shot length (sec)	0.17	1	0.015	0.05	0.011
Maximum shot length (sec)	41	34	16	27	101

Table 2 shows that the ASL and MSL values for *The Seventeenth Abandonment*, *Tomorrow is Another Day*, and *Open Your Heart* are relatively close, indicating a similar intensity of editing in terms of ASD individuals emotional characteristics. In contrast, *He is My Brother* displays a significant numerical difference, with a 1.9 s gap between its MSL and ASL, suggesting considerable variability in the shot lengths throughout the films. As shown in Table 2, the long shot in *He is My Brother*, which depicts the older brother, Guang Wen, securing a piano tuning job with his musical talent while walking down a wide street with his younger brother, Xiu Wen, discussing their future, is the longest shot among the five films. This shot significantly influences the normal distribution of shot lengths in the entire film. Through this long shot, the roles remain immersed in the emotional atmosphere created by the scene, guiding the public to focus on the subtle details of their expressions and movements, thus conveying the profound bond between two brothers. Not only does this shot emphasize their close relationship, but it also symbolizes their hope for the future.

In Figures 3, a comparison of the peaks and valleys reveals noticeable differences in the distribution of shot lengths and editing rates across autism-themed films from different years. In *Tomorrow is Another Day*, the emotional characteristics of the ASD individuals protagonist are especially prominent; the autistic protagonist exhibits a noticeable lack of adaptive strategies, with clear emotional disturbances resulting in a slightly faster editing pace compared to the other films.

### 4.2 Editing rate, motion shots, and transitions based on film rhythm analysis

In film rhythm analysis, editing rate, motion shots, and transitions are three normally utilized measurements. In this paper, we use them to investigate the rhythmic patterns and emotional impacts within a selection of autism-themed films.

#### 4.2.1 Editing rate-based rhythm analysis

**Figure 4:**
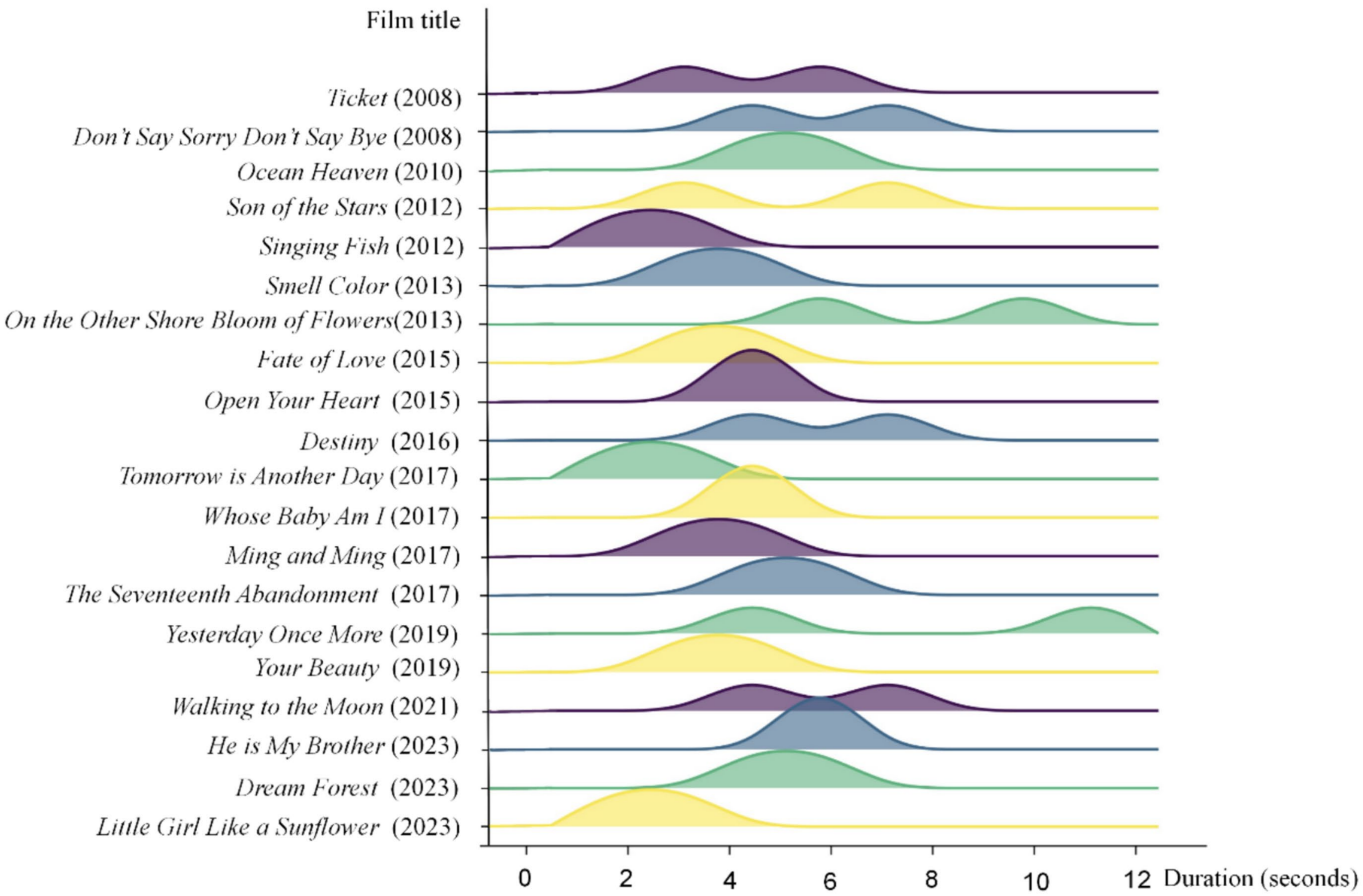
Comparison of ASL and MSL of emotional characteristics clips of 20 autism-themed films.

The length of its shots determines the rhythm of a film. Longer ASL results in a lower editing rate (or frequency) and a slower rhythm, while shorter ASL corresponds to a higher editing rate and a faster rhythm (26, 27). In the book *Rhythm in Film* by D. W. Griffith, he emphasizes that rhythm is more crucial than both stars and the storyline in films that profoundly touch the heart. Rhythm is central to the art of filmmaking and must be treated with the utmost seriousness, not perfunctorily (38). In *On Cinematic Rhythm* by Léon Muscinak, he states that rhythm stems from the need for a psychological dimension that complements and completes spatial and temporal perception through the unconscious activity of the human being (39). However, it is important to note that extremely long or short clips may skew the data set. Therefore, ASL alone does not fully capture the dynamic nature of film styles. In particular, when there are frequent outliers, ASL is better suited as a reference for comparative style analysis rather than as a tool for determining overall trends (40). In order to analyze the rhythm of 20 selected films, a comparison of ASL and MSL is carried out in this paper, where Table 3 shows the comparison data between ASL and MSL for the emotional characteristics clips of the 20 autism-themed films. Furthermore, Figure 4 visualizes the difference between ASL and MSL through the peaks and valleys graph, which together form the fundamental analysis of the editing rate of the film.

**Table 3:** Comparison of ASL and MSL for emotional characteristics of clips of 20 autism-themed films, where “sec” represents “second.”

Title (release date)	ASL (sec)	MSL (sec)
*Ticket* (2008)	6.1	3
*Do not Say Sorry Do not Say Bye* (2008)	6.9	4
*Ocean Heaven* (2010)	5.6	4
*Son of the Stars* (2012)	7.4	3.5
*Singing Fish* (2012)	3.0	2
*Smell Color* (2013)	4.4	3
*On the Other Shore Bloom of Flowers* (2013)	10.3	6
*Fate of Love* (2015)	4.4	3
*Open Your Heart* (2015)	4.9	4
*Destiny* (2016)	7.5	5
*Tomorrow is Another Day* (2017)	3.0	2
*Whose Baby Am I* (2017)	4.2	4
*The Seventeenth Abandonment* (2017)	5.5	5
*Ming and Ming* (2017)	4.8	3
*Your Beauty* (2019)	4.3	3
*Yesterday Once More* (2019)	10.9	5
*Walking to the Moon* (2021)	7.6	5
*He is My Brother* (2023)	5.6	6
*Dream Forest* (2023)	5.8	4
*Little Girl Like a Sunflower* (2023)	2.6	2

Combining the editing rates in Table 3 and Figure 4, it can be seen that the editing rates of *Singing Fish* and *Tomorrow is Another Day* are the same, both are 3.0 s, and in comparison, *Little Girl Like a Sunflower* is 0.36 s faster than *Singing Fish* and *Tomorrow is Another Day*. The editing rate of these three films is significantly faster than others in these 20 films, which reflects that the actions are repetitive, with more pronounced avoidance and emotional disturbances, and a distinctive presentation of the emotional characteristics of ASD individuals in these films. To help the public better understand and feel the emotional fluctuations of the ASD roles, the films effectively direct the attention of the public by speeding up the editing rhythm, reinforcing the transmission of the emotional changes of the roles, thus triggering deeper emotional resonance in the minds of the public and eliciting the experience of heart flow.

This rhythm not only adds tension to the film but also allows the public to feel the inner world of the roles more intuitively in a short period. In comparison, the editing rate of the films *Ocean Heaven* and *Dream Forest* fluctuates up and down at 5.62 s. The difference in ASL between the two is only 0.18 s, but the editing rate of *Ocean Heaven* is slightly faster, which may stem from the recurring hand motions of the ASD individuals protagonist in the film, as well as the special fascination with rotating objects. These types of movements are presented throughout the entire film, causing the film to select a faster editing rate in certain clips to reflect the stereotypical behaviors and psychological traits of the roles, thus increasing the emotional tension of the film.

From the analyses of Table 3 and Figure 4 above, we can find that these films are meticulously crafted in terms of editing rhythm, not only to serve the emotional expression of roles within the plot but also to profoundly depict the emotional traits and behavioral patterns of individuals with ASD through shifts in visual language. In this way, the films more effectively guide the public to empathize with the emotional states of the ASD roles, thereby fostering greater understanding and tolerance of this unique group.

#### 4.2.2 Motion shots based rhythm analysis

**Figure 5:**
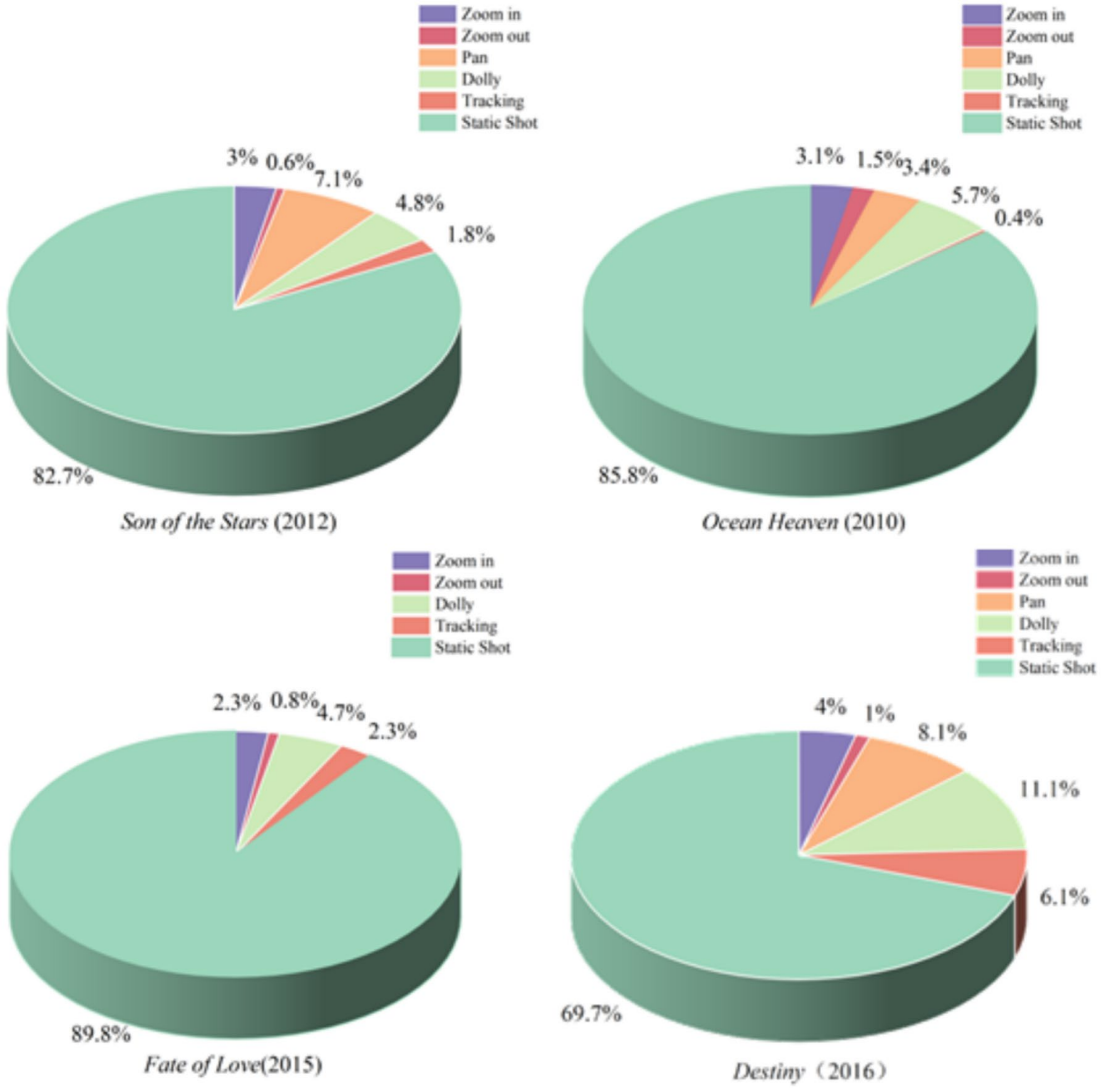
Pie chart for the shooting methods of ASD emotional characteristics.

Motion shots refer to a filming technique where the camera itself is in movement during the exposure, and it primarily creates a dynamic and fluid perspective to follow action, reveal information, explore spaces, or immerse viewers in the narrative. Motion shots also serve as a key metric for measuring visual rhythmic flow in cinemetrics, where motion shots and editing rhythm are usually used to determine the visual presentation and emotional rhythm in a film, jointly (26, 27). In the following part, the motion shots and transitions in four films are analyzed, including *Ocean Heaven*, *Son of the Stars*, *Fate of Love*, and *Destiny*. To this end, Figure 5 presents a pie chart depicting the shooting style of emotionally charged clips featuring ASD individuals’ roles, offering intuitive data support for examining the role of motion shots in emotional expression and narrative rhythm.

The data reveals that *Destiny* employs a significantly higher number of motion shots using techniques such as zoom in, zoom out, pan, dolly, and tracking compared to *Ocean Heaven*. The total number of motion shots in *Son of the Stars* also surpasses that in the microfilm *Fate of Love*, with *Destiny* featuring 30% of total camera movements, 15.6% more than *Ocean Heaven*, and 16.6% in *Son of the Stars*, which is 6.5% higher than that in *Fate of Love*. These data reflect that the use of motion shots in films not only serves a visual function but also reflects the emotional states of the characters, particularly in expressing the emotional fluctuations of ASD individuals characters. In *Destiny*, camera movements predominantly express the emotional characteristics of ASD individuals through horizontal dollying and panning shots. It conveys the emotional instability and continuous changes experienced by individuals with ASD. Individuals with ASD often exhibit discontinuous emotional fluctuations in their emotional expression, and the use of these shots precisely reflects the instantaneous changes and ongoing fluctuations of ASD individuals emotions. In contrast, in *Fate of Love*, the primary camera movements are panning, zooming in, and tracking shots. This contrast highlights the differences in how the two films narrate the emotional traits of ASD individuals. The comparison further underscores the need for increased rehabilitation training for children and adolescents with ASD individuals, calling for special support from the general education system, and reinforcing the importance of strengthening special education training for general teachers. “The Second Special Education Enhancement Program (2017–2020)” explicitly states that it is a collective societal responsibility to provide appropriate education for children with disabilities, including those with ASD individuals (41). Additionally, *Destiny* immerses the public in the narrative through dynamic camera movements, such as dolly and pan shots, enabling a deeper understanding of the emotional fluctuations experienced by ASD roles. The multi-layered narrative structure of the film further enhances this effect: alongside the main storyline of Xihe and his mother, the film weaves in two subplots involving Xihe’s classmate Zixiang and his grandmother, as well as Xihe’s uncle and grandmother. The parallel portrayals of these three groups of ASD roles spanning childhood, adolescence, and middle age add depth and complexity to the narrative of the film (42).

#### 4.2.3 Transitions-based rhythm analysis

**Figure 6:**
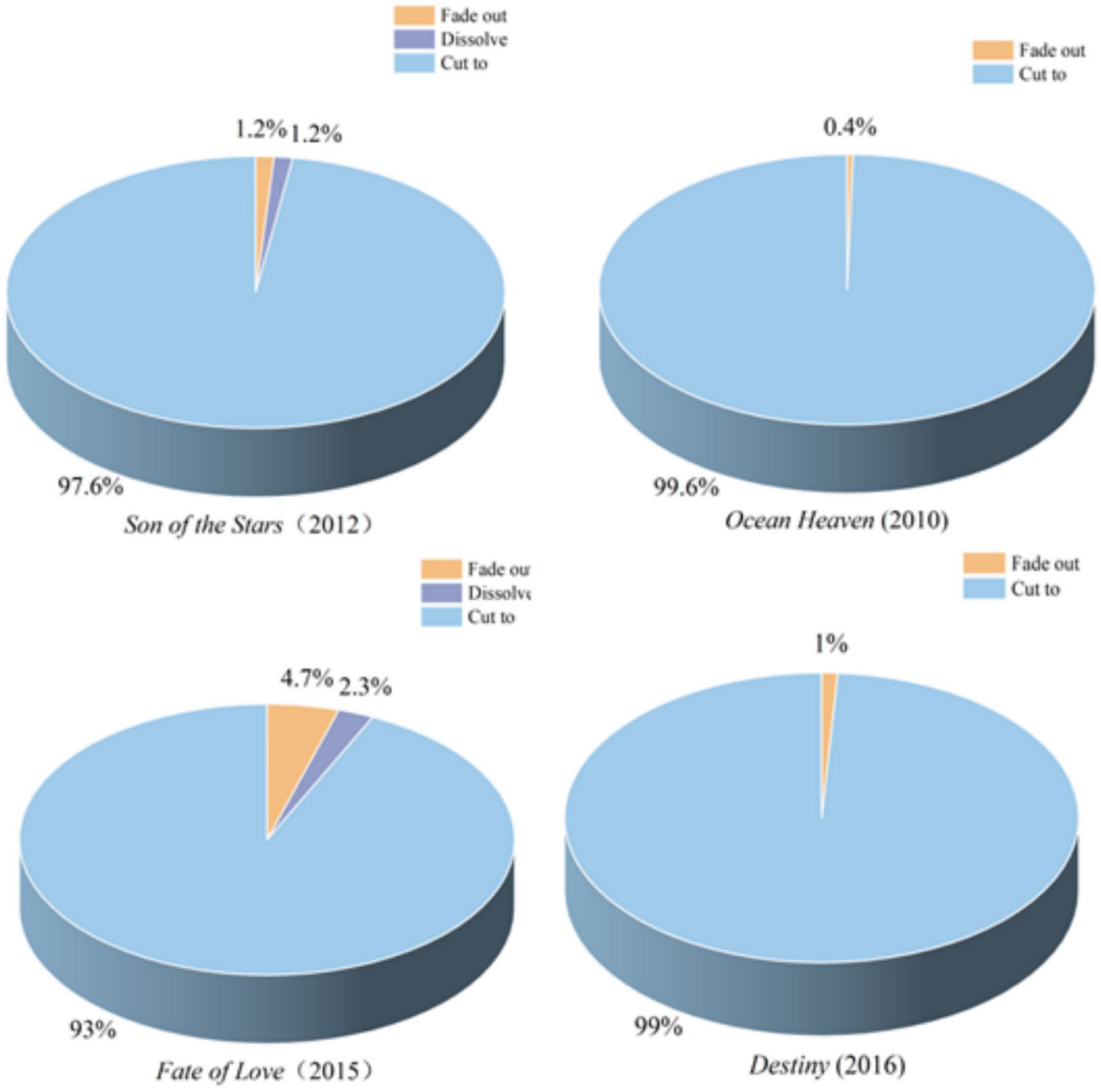
Pie chart for the transition methods of ASD emotional characteristics.

A film transition is a filmmaking technique in shots or scenes, defines the connection between narrative units, controls the pace and rhythm in films, and serves as a powerful tool for conveying emotion, meaning, and the passage of time (26, 27). In Figures 6, a pie chart illustrates the transitions in four ASD-related emotional characteristics clips.

The data in Figure 6 indicate that dissolve and fade out are used significantly more frequently in *Son of the Stars* than in *Ocean Heaven*. Furthermore, in *Fate of Love*, the frequency of these two transitions is notably higher compared to *Destiny.* Specifically, the use of dissolve and fade out is 2% higher in *Son of the Stars* than in *Ocean Heaven*, and 6% in *Fate of Love* than in *Destiny*. These data suggest a substantial difference in the application of dissolve and fade out in films when conveying ASD-related emotions. As important editing techniques, these transitions serve to link and guide the narrative. They not only facilitate a smoother storytelling experience for the public but also deepen their understanding of the emotional characteristics of ASD individuals. Through these techniques, the public can experience more profoundly the inner changes and emotional complexities of ASD individuals.

### 4.3 Shot types based on film space analysis

**Figure 7:**
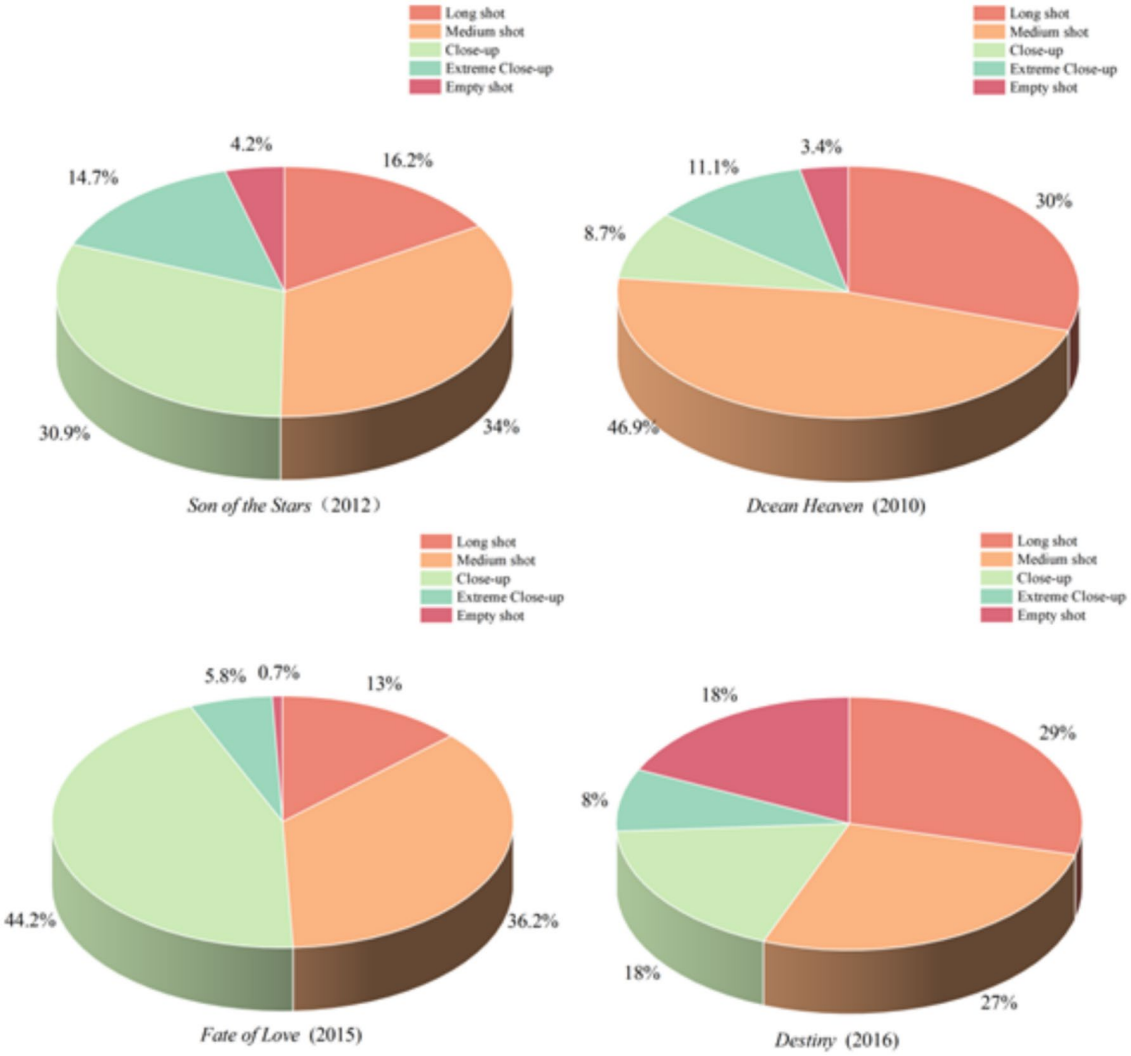
Pie chart of shot type distribution of ASD emotional characteristics.

Shot type is one of the most fundamental tools in filmmaking, which directs the attention of the public and reveals the emotions of roles in films. Shots are primarily categorized by how much of the subject and surroundings we see, including extreme long shot, long shot, medium shot, close-up, extreme close-up, and empty shot (26, 27). Shot type and spatial composition within the lens are essential components of cinematic language. Shot type transitions not only shift the viewpoint but also regulate and restrict the field of vision of the viewer, thereby determining the visual information they receive. This change guides the public to focus on different aspects of the subject, endowing the image with a sense of hierarchy, emphasis, and order, thus making the purpose and direction of the image expression more explicit (43). To enhance the visual language, films often employ various technical means, such as increasing the frequency of cuts, utilizing extremely long or short focal length shots, and incorporating a large number of close-up solo shots (44). As shown in Figure 7, four films are selected in this paper for analyzing shot types, including *Son of the Stars*, *Ocean Heaven*, *Fate of Love*, and *Destiny*.

Figure 7 shows a pie chart depicting the distribution of shot types within emotionally significant clips in four films. The results indicate that long shot, medium shot, and close-up are the most commonly used, collectively accounting for over 70% of emotional clips in each film. Specifically, *Son of the Stars* and *Fate of Love* predominantly employ medium shots and close-ups, whereas *Ocean Heaven* and *Destiny* prefer to use medium and long shots. These various shot types are applied to represent different emotional characteristics of ASD individuals. Among these four films, *Ocean Heaven* exhibits the highest proportion of long shots, medium shots, and close-ups, which comprise 85.6% of its emotional characteristics in total.

This film explores the future of children with ASD individuals after they grow up, through the theme of entrusting a child to someone else before death. In the film, Dafu’s father not only teaches him self-care abilities, but also gradually helps Dafu master basic life skills through patient and repeated instruction. After the passing of his father, Dafu benefits from this training, and it demonstrates that individuals with ASD can develop greater independence in a supportive and nurturing environment. This storyline not only highlights the profound love of a parent but also raises broader concerns regarding the long-term well-being of individuals with ASD.

In contrast, *Destiny* features the highest proportion of empty shots, which account for 18% emotional characteristics in total. These empty shots not only visually create white space but also carry rich connotations and profound meanings within the context of the film. Despite the absence of tangible physical elements within the frames, the emotions and symbolic meanings they convey are diverse and intricate (45). The emotionally significant clips in *Destiny* center on three groups of ASD roles, addressing critical social issues such as challenges in education and sex education for adolescents with ASD individuals. In these sequences, empty shots serve as a powerful narrative tool, encouraging deeper public reflection on the unique needs and struggles of individuals with ASD. Through this technique, the film not only portrays the inner world of its characters but also fosters greater social awareness and emotional resonance.

### 4.4 Statistical analysis of cinemetrics indices

*Significant Difference* is a fundamental statistical criterion used to determine whether observed variations between samples are statistically meaningful (46). The Paired Samples *t-*test is a statistical method specifically designed to assess whether a significant difference exists between two related samples. In this paper, to investigate the rhythmic features of emotional expression in roles portrayed in autism-themed films, 20 representative films are selected. For each scene, ASL and MSL are both calculated. As both ASL and MSL are derived from the same film clips and represent two dimensions of the same dataset, they constitute paired observations. Accordingly, the Paired Samples *t*-test is employed to examine whether there exists a statistically significant difference between the two measures. Paired Samples *t*-test is defined in Equation 1:


(1) $$t=\frac{\bar{d}}{S_{d}/\sqrt{N}}$$

In Equation 1, $\bar{d}$ represents the mean of the paired differences (i.e., the difference between ASL and MSL for each pair); $S_{d}\;$is the standard deviation of the differences; N denotes the number of paired samples (N=20 in this paper); tis the test statistic. Furthermore, in a Paired Samples *t*-test, if the significance level (*p-*value) is below 0.05 or 0.01, then the difference is considered statistically significant or highly significant, respectively. In this paper, Python is applied to calculate the corresponding *t* and *p-*value (= 0.05 in this paper), where the paired samples *t*-test comparing average ASL and MSL in emotional characteristics clips of autism-themed films is shown in Table 4.

**Table 4:** Paired samples *t*-test for 20 autism-themed films.

Pairing difference						*t*	Freedom	Significance	
	Mean difference	Standard deviation	Standard error mean	95% confidence interval					
				Lower limit	Upper limit			Unilateral *p*	Bilateral *p*
Pair1 ASL-MSL	1.980 s	1.526	0.341	1.204	2.632	5.620	19	<0.001	<0.001

**Figure 8:**
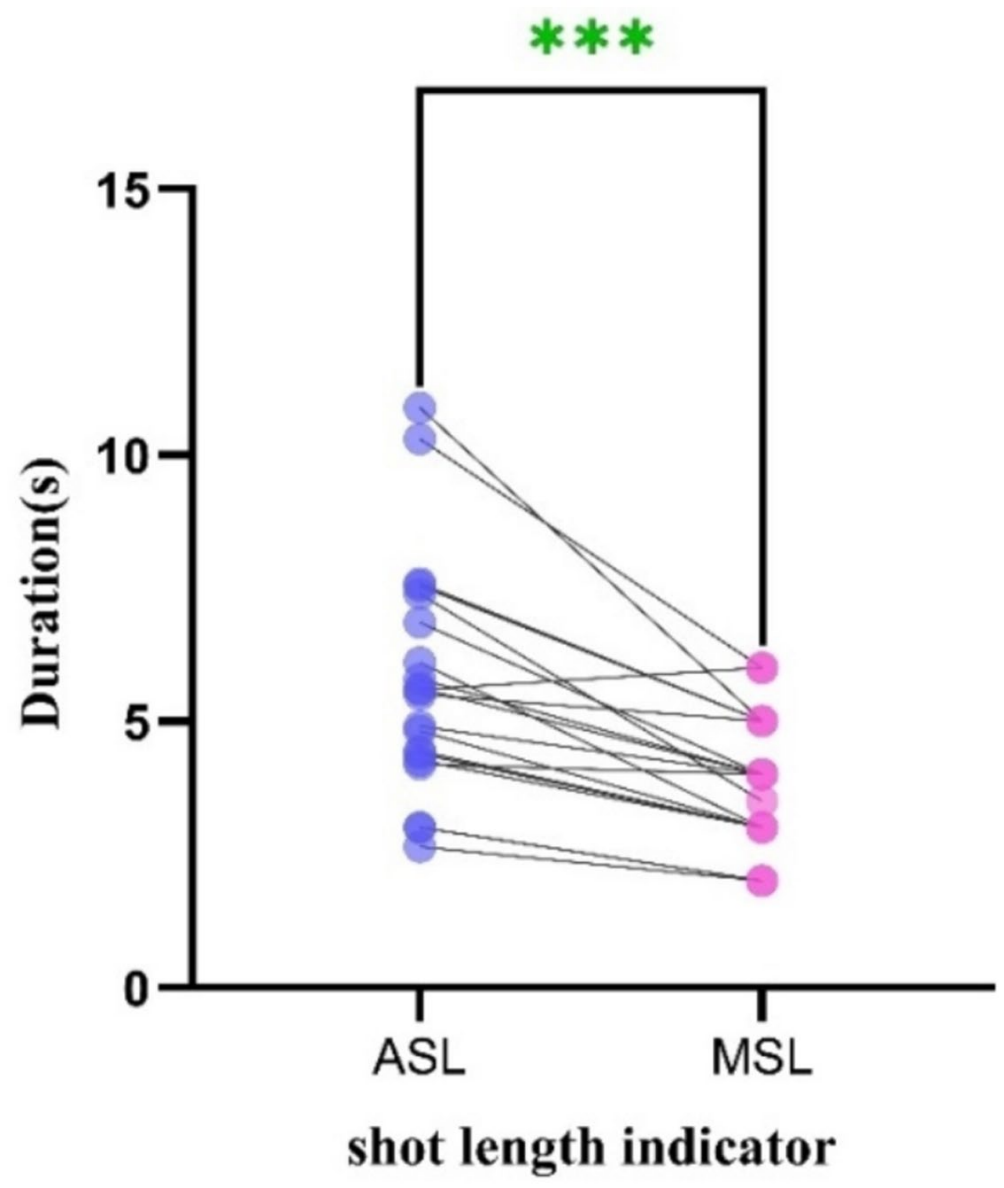
Normality test for 20 autism-themed films.

Table 4 shows the mean difference (ASL–MSL), standard deviation, standard error of the mean, 95% confidence interval, *t* statistic, degrees of freedom, and significance levels for both one-tailed and two-tailed tests. Across 20 autism-themed films, ASL is on average 1.980 s longer than MSL (standard deviation = 1.526), with a 95% confidence interval of [1.204, 2.632]. The paired *t*-test indicated that this difference was highly significant, *t*(19) = 5.620, *p* < 0.001 (bilateral). Furthermore, the results of the normality test are shown in Figure 8, where each line connects paired ASL and MSL values from the same film, indicating that ASL values are generally higher than MSL values.

In Figure 8, the green asterisks (***) denote a highly significant difference between ASL and MSL (*p* < 0.001). Taken together, these results demonstrate that shot durations measured by ASL are significantly longer than those measured by MSL, and that the data meet the normality assumption required for the paired *t*-test. Consequently, indicators such as ASL and MSL can serve as effective tools for capturing and quantifying the emotional rhythm of on-screen ASD roles. Through an in-depth examination of these rhythmic and stylistic patterns, filmmakers can achieve a more authentic portrayal of the ASD individuals emotional experience, thereby cultivating deeper understanding and empathy in the public.

This paper employs a Paired Samples *t*-test to analyze the differences between ASL and MSL, finding these differences to be statistically significant (*p* < 0.001). However, the results of the Paired Samples *t*-test primarily reveal the differences between ASL and MSL without delving into the specific relationship between these differences and emotional characteristics. Therefore, the interpretation of the connection between ASL-MSL differences and emotional traits remains somewhat speculative.

### 4.5 Discussion

#### 4.5.1 Strength of this paper

Analysis of film data from 2008 to 2024 indicates a consistently higher ASL than MSL, a trend indicative of the evolving nature of cinematic works. This shift challenges the previous tendency in films to overly alienate and dramatize the portrayal of individuals with ASD. It emphasizes that individuals with ASD are not merely exceptional cases with extraordinary talents but represent a broader group that exists within society. Nowadays, filmmakers have become more adept at accurately depicting the emotional characteristics of individuals with ASD, presenting portrayals that are increasingly aligned with the real-life experiences of individuals with ASD. This enables audiences to gain a deeper understanding of and concern for this group.

The visual style and rhythm of films are not merely tools for artistic expression but serve as important media for conveying the psychological states and behavioral challenges of individuals with ASD. Through techniques such as shot length, editing rhythm, and camera movement, films effectively depict the unique emotional regulation, social interaction, and social cognition of individuals with ASD. These portrayals are closely linked to psychological concepts such as emotional regulation, theory of mind, and social cognition. Therefore, the classic theories of theory of mind by Baron-Cohen et al. (47), as well as Happé’s (48) exploration of social cognition, provide us with a multidimensional perspective that aids in understanding the emotional and behavioral expressions of individuals with ASD in films.

These studies indicate that the portrayal of ASD individuals in films is not merely a visual and emotional representation but also a reflection of psychological mechanisms, which deepens our understanding of individuals with ASD. Notably, the shorter the ASL, the more pronounced the emotional characteristics of ASD individuals appear. This phenomenon suggests that the rhythm and shot composition in films not only affect the conveyance of emotions but also reflect the unique nature of emotional expression in individuals with ASD. It further emphasizes the profound impact that films have in shaping public perceptions of ASD individuals.

#### 4.5.2 Limitations of this paper

Due to the lack of a control group for non-ASD films, we cannot definitively determine whether the ASL-MSL pattern is limited to autism-themed films. Therefore, our current results in the paper primarily reflect the emotional characteristics found in Chinese autism-themed films and do not apply to a broader comparison across different film genres. To enhance the generalizability and comparability of this paper, we consider incorporating non-ASD films as a control group for cross-genre comparative analysis in the future. This will facilitate a more comprehensive assessment of whether the shot lengths and pacing patterns observed in autism-themed films also exist in other film genres, and whether these patterns have universal applicability or are specific to the emotional characteristics of ASD.

Furthermore, the scope of this paper is limited to Chinese films and thus does not comprehensively reflect the global portrayal of ASD individuals. To address this limitation, we are conducting further research on the analysis of autism-themed films from other countries. We aim to extend these preliminary findings to films from different countries in order to explore the universality and cultural differences in the emotional characteristics of ASD individuals across global cinema.

Besides, the current descriptive statistical results only support the conclusions that can be drawn within the scope of the available data. In future research, we plan to employ more advanced statistical methods, such as multivariate analysis or regression analysis, to explore further how ASL-MSL differences relate to emotional characteristics (e.g., emotional fluctuations, emotional intensity). This will help provide a more precise interpretation of the emotional traits reflected in the ASL-MSL differences and avoid relying on single, speculative interpretations.

#### 4.5.3 Suggestions for ASD intervention

Based on the cinemetrics analysis results of autism-themed films in Section 4, we propose three intervention ways for ASD individuals:

##### 4.5.3.1 Visual presentation technique-based personalized intervention planning

Based on the visual presentation of emotional characteristics of ASD individuals in cinemetrics, we can develop personalized intervention plans for individuals with ASD. For those with difficulties in recognizing expressions, practitioners can refer to the visual data of expression changes in films and develop expression training plans, such as guiding them to recognize and imitate expressions by watching specific film clips (49). For individuals with ASD rigid body movements, practitioners can design corresponding behavioral modification training based on the visual presentation of changes in movement frequency and amplitude, using games or activities to gradually guide them to reduce rigid movements and increase flexible body movements.

##### 4.5.3.2 Computer vision-based emotion recognition and regulation training

Based on computer vision techniques, practitioners can utilize the visual resources of film footage to conduct emotion recognition and regulation training for individuals with ASD. Practitioners can edit out clear emotional expression clips from films and turn them into training materials. During the training process, with the help of visual tools such as expression recognition software, ASD individuals can compare their expressions with those of film roles to learn to recognize different emotions. Meanwhile, by analyzing the emotional regulation methods of roles in films under different situations, ASD individuals can be guided to learn how to cope with their emotional changes.

##### 4.5.3.3 Visualization technique based on interacting with virtual roles

Based on virtual reality (VR) or augmented reality (AR) techniques, practitioners recreate film scenes and immerse ASD individuals in them, allowing them to interact with virtual roles. Through the visualization technique, real-time feedback is provided on the emotions and behavioral expressions of ASD individuals during interaction, and practitioners provide timely guidance based on the feedback information. For example, in a simulated social scene of supermarket shopping, ASD individuals can attempt to communicate with a virtual cashier based on the social behavior patterns of roles in the film. Practitioners evaluate and improve the social performance of patients by observing visual data such as the duration of eye contact and the fluency of language expression.

It can be seen that combining the visual analysis of cinemetrics with visualization techniques can provide a new thought for the intervention of individuals with ASD that is both scientific and practical. This thought not only precisely meets the core needs of ASD individuals in areas such as emotion recognition, physical behavior, and social interaction, but also reduces the threshold of intervention through personalized training and immersive experiences. Furthermore, it breaks the limitation of traditional intervention models, which often feature more abstract guidance and fewer concrete scenarios. It provides a more valuable practical paradigm for special education and psychological intervention research, allowing the integration of technology and cinemetrics to truly serve every individual in need.

## 5 Social significance of autism-themed film studies

According to the “Seventh National Population Census of the People’s Republic of China and survey data from the China Disabled Persons’ Federation (2021),” the largest population of persons with disabilities globally is in China. The five major disability groups, visually impaired, hearing and speech impaired, physically impaired, intellectually disabled, and mentally challenged, comprise over 85 million people, accounting for approximately 6.34% of the total population. This translates to one in every 16 individuals having a disability. As Leonard Davis asserts, disability is a dynamic condition (50). Even those who are currently able-bodied may experience disability due to disease, aging, or functional deterioration. He emphasizes, while racial identity and sexual orientation are largely fixed aspects of one’s being, the state of being able-bodied is not a permanent guarantee. Disability is a category that any person might enter due to an accident, illness, or simply the process of aging. From this perspective, addressing the needs of people with disabilities is not merely an act of social responsibility but also a reflection on the potential future of every individual. Films, as a distinct form of artistic expression, can vividly depict the lived experiences of people with disabilities, fostering a deeper understanding of their challenges and perspectives. At the group meeting of members from the cultural, artistic, and social science circles during the “Second Session of the Thirteenth National Committee of the Chinese People’s Political Consultative Conference (CPPCC),” President Xi Jinping emphasized that all literary and artistic works and academic research of great value and significance should reflect and engage with reality to contribute to solving practical problems and addressing real-world challenges. Literary and artistic creation should be grounded in local realities, deeply embedded in the spirit of the times, and committed to enhancing the spiritual depth, cultural significance, and artistic value of the works (51).

**Figure 9:**
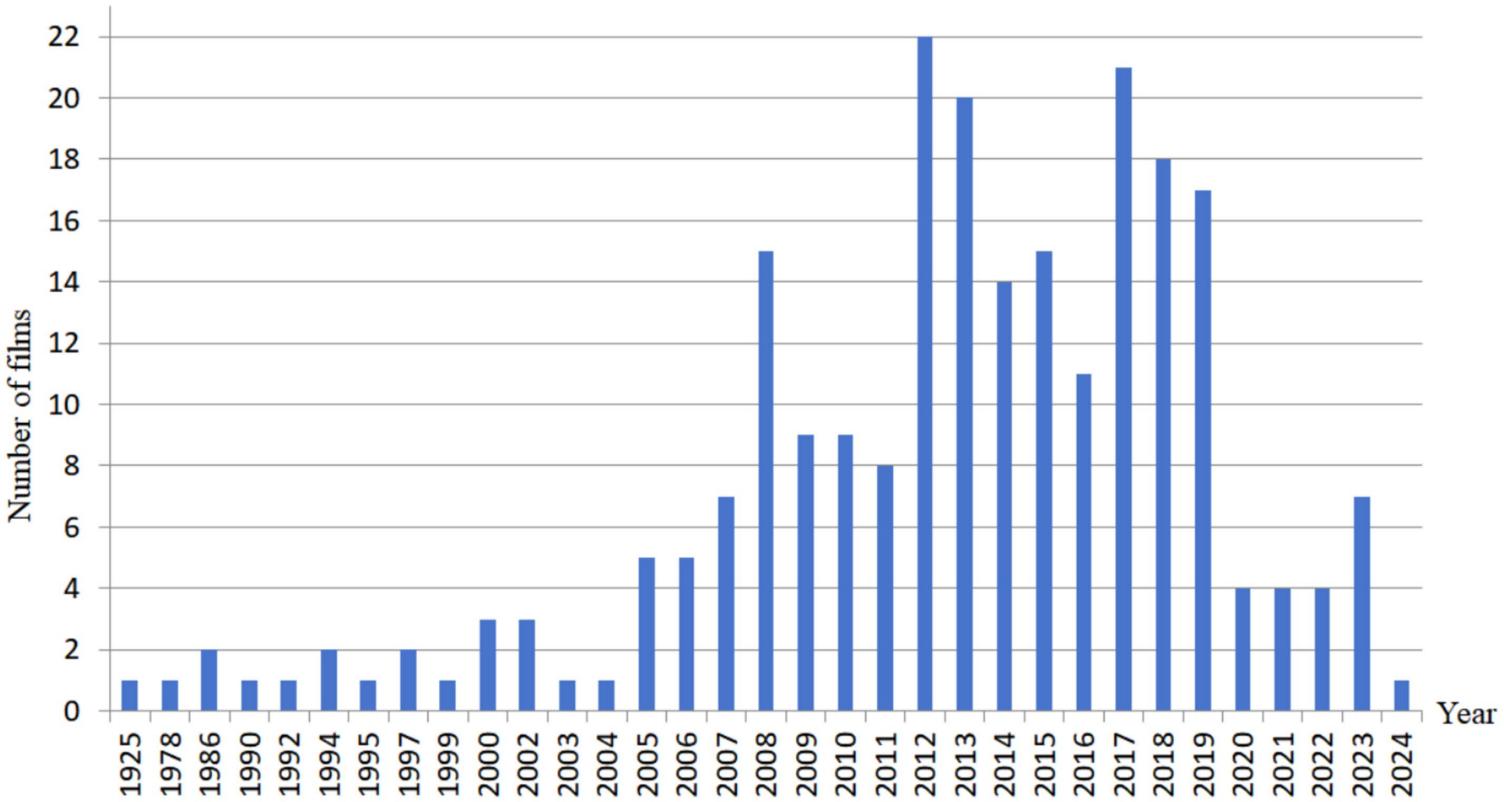
Histogram of the number of Chinese films with disability themes.

This paper analyzed 20 Chinese films depicting the emotional characteristics of individuals with ASD. However, this represents only a small subset of disability-themed films. By reviewing ratings from the Chinese Cinema Knowledge System platform, China Film Network, IMDB, and other online film databases, as well as relevant literature, a total of 236 disability-themed films produced between 1925 and 2024 were identified. Figure 9 shows a histogram illustrating the distribution of these films over time, revealing a significant increase in their production after 2008, peaking in 2012, and stabilizing thereafter.

In Figure 9, the trend is closely linked to national policy developments. Historically, individuals with disabilities in China were often stigmatized with negative labels such as “karma,” “useless,” and “invalid.” However, with the rise of civil rights and social movements, the concept of respecting the human rights of persons with disabilities has gained traction. In 2008, China amended and enacted the “Law of the People’s Republic of China on the Protection of Persons with Disabilities,” which explicitly guarantees that persons with disabilities enjoy the same rights as other citizens in various domains, including political participation, economic activity, cultural engagement, social integration, and family life. Their citizenship rights and human dignity are strictly safeguarded by national law (52). Further reinforcing these protections, in 2012, the General Office of the State Council issued the Circular on the Outline of the Development Program for Poverty Reduction for Persons with Disabilities in Rural Areas (2012–2020) (53), which strengthened the legal and policy framework for safeguarding the rights and interests of persons with disabilities.

With the continuous introduction and refinement of relevant policies, public awareness of individuals with disabilities has progressively increased. In addition to policy advancements, technological innovations have significantly contributed to enhancing the quality of life for people with disabilities. In 2019, Tapu et al. developed the Deep-Hear system (54), which integrates face recognition, video segmentation, speech recognition, and subtitle recognition technologies. This system labels subtitle text near the speaker, thereby improving video accessibility for individuals with hearing impairments. Simultaneously, the number of films addressing disability-related themes has grown substantially. This trend has not only heightened societal awareness of individuals with disabilities but also reflects a broader shift from discrimination and prejudice toward respect and understanding. Such films serve as a medium for fostering public recognition of the disability community while also deepening the understanding of the public of the emotional characteristics of individuals with conditions such as ASD. By promoting inclusivity and empathy, these films contribute to a more equitable and respectful society, benefiting both individuals with disabilities and their families.

## 6 Conclusion and future work

This paper uses the cinemetrics method to analyze emotional segments in 20 Chinese autism-themed films quantitatively, examining differences in shot length, editing rhythm, and visual style. The findings indicate that the pacing and filming techniques in these films effectively reflect the unique emotional regulation and social cognition of individuals with ASD. Faster editing and shorter shots depict emotional fluctuations, while longer shots highlight introspective thoughts and emotional suppression. The films balance close-ups and medium shots to allow the audience to feel the emotional changes of autistic characters and observe their condition, fostering emotional resonance. Through data visualization and analysis, the study offers a more objective and dynamic approach to exploring emotional characteristics, enhancing the practical value of the research.

The paper confirms that the ASL is generally greater than the MSL, reflecting systematic variations in emotional characteristics and rhythm in autism-themed films. Although the sample is limited to Chinese films, the findings offer valuable insights into the emotional expression of individuals with ASD. To address this limitation, we are expanding to include films from other countries and have already built up an expression database for autism-themed films from regions outside China (55). Furthermore, in order to enhance the objectivity of the data preparation method, we have also developed a deep learning based ASD expression detection approach, which can improve the accuracy and applicability of emotional expression analysis (28). The integration of film metrics and visualization technologies offers a new scientific framework for ASD intervention, providing effective, personalized training methods to support individuals with ASD.

## Data Availability

The original contributions presented in the study are included in the article/supplementary material, further inquiries can be directed to the corresponding author/s.

## Footnotes

^1^The 20 films include *Do not Say Sorry Do not Say Bye* (Jiankui Liu, 2008), *Ticket* (Zhiliang Zhang, 2008), *Ocean Heaven* (Xiaolu Xue, 2010), *Son of the Stars* (Miao Chen, 2012), *Singing Fish* (Shu San, 2012), *Smell Color* (Xiubo Hao, 2013), *On the Other Shore Bloom of Flowers* (Jia Qi, 2013), *Open Your Heart* (Zhen Yang, 2015), *Fate of Love* (Tao Shi, 2015), *Destiny* (Wei Zhang, 2016), *Whose Baby Am I* (Nan Li, 2017), *Tomorrow is Another Day* (Dali Chen, 2017), *Ming and Ming* (Haobo Hu, 2017), *The Seventeenth Abandonment* (Zhuolin Li, 2017), *Yesterday Once More* (Qidong Zhang, 2019), *Your Beauty* (Li Xu, 2019), *Walking to the Moon* (Yangqing Chen, 2021), *Little Girl Like a Sunflower* (Yingqi Chen, 2023), *He is My Brother* (Xiuzhuan Guo, 2023), *Dream Forest* (Chao Fan, 2023).
